# An explorative investigation of functional differences in plantar center of pressure of four foot types using sample entropy method

**DOI:** 10.1007/s11517-016-1532-7

**Published:** 2016-06-16

**Authors:** Zhanyong Mei, Kamen Ivanov, Guoru Zhao, Huihui Li, Lei Wang

**Affiliations:** 1 College of Information Science and Technology, Chengdu University of Technology, Chengdu, People’s Republic of China; 2 Shenzhen Institutes of Advanced Technology, The Shenzhen Key Laboratory for Low-cost Healthcare, 1068 Xueyuan Avenue, Shenzhen University Town, Shenzhen, 518055 People’s Republic of China; 3 Graduate University of Chinese Academy of Sciences, Beijing, 100049 People’s Republic of China

**Keywords:** Gait, Foot type, Plantar pressure, Biomechanics

## Abstract

In the study of biomechanics of different foot types, temporal or spatial parameters derived from plantar pressure are often used. However, there is no comparative study of complexity and regularity of the center of pressure (CoP) during the stance phase among pes valgus, pes cavus, hallux valgus and normal foot. We aim to analyze whether CoP sample entropy characteristics differ among these four foot types. In our experiment participated 40 subjects with normal feet, 40 with pes cavus, 19 with pes valgus and 36 with hallux valgus. A Footscan^®^ system was used to collect CoP data. We used sample entropy to quantify several parameters of the investigated four foot types. These are the displacement in medial–lateral (M/L) and anterior–posterior (A/P) directions, as well as the vertical ground reaction force of CoP during the stance phase. To fully examine the potential of the sample entropy method for quantification of CoP components, we provide results for two cases: calculating the sample entropy of normalized CoP components, as well as calculating it using the raw data of CoP components. We also explored what are the optimal values of parameters *m* (the matching length) and *r* (the tolerance range) when calculating the sample entropy of CoP data obtained during the stance phases. According to statistical results, some factors significantly influenced the sample entropy of CoP components. The sample entropies of non-normalized A/P values for the left foot, as well as for the right foot, were different between the normal foot and pes valgus, and between the normal foot and hallux valgus. The sample entropy of normalized M/L displacement of the right foot was different between the normal foot and pes cavus. The measured variable for A/P and M/L displacements could serve for the study of foot function.

## Introduction

**Fig. 1:**
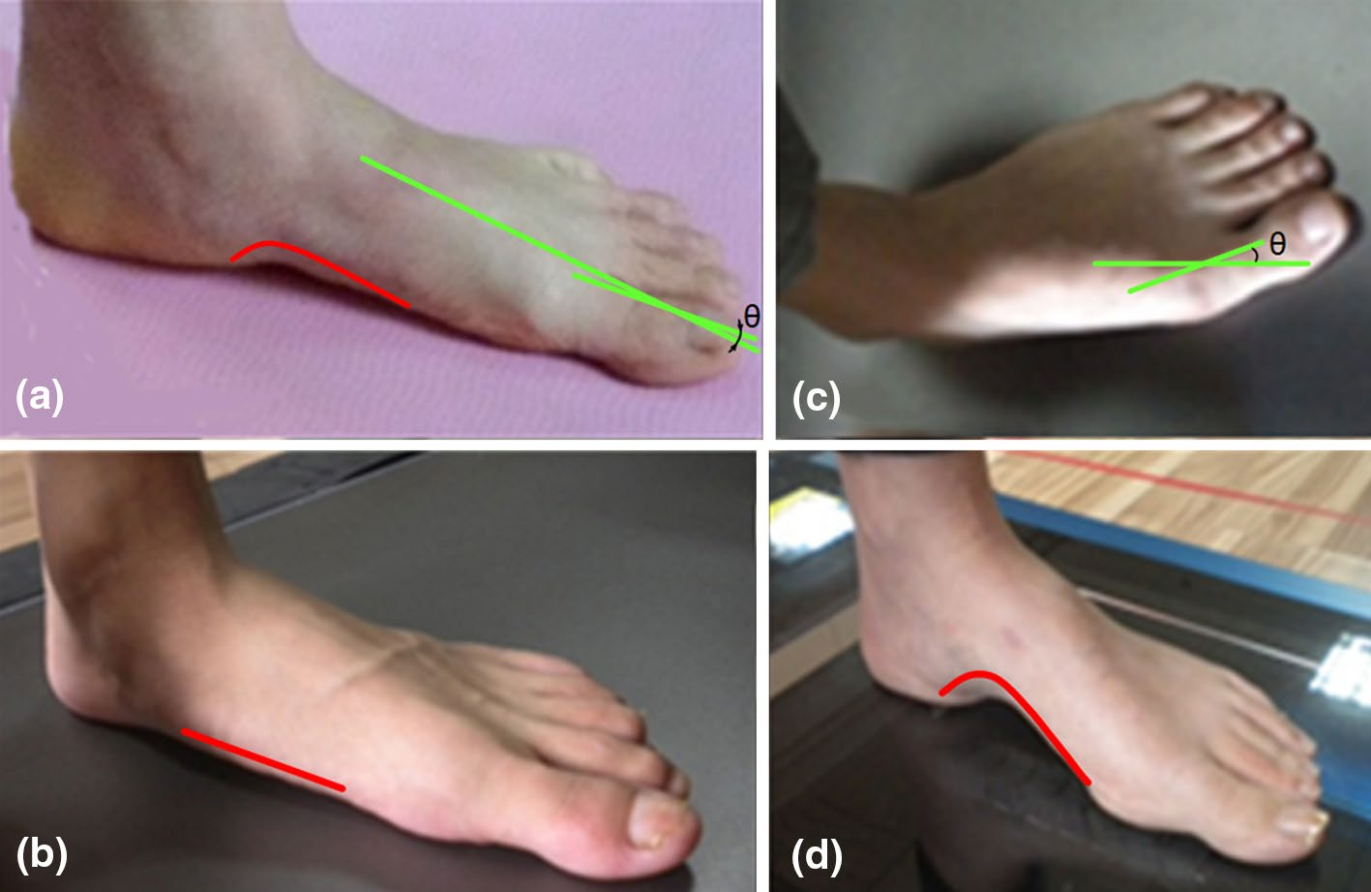
**a** Normal foot, **b** pes valgus, **c** hallux valgus, **d** Pes cavus. The foot arches are illustrated with *red lines*. Pes valgus is characterized by a collapse of longitudinal arches while pes cavus appears with abnormally high medial longitudinal arches. The first metatarsophalangeal angle is marked with *green lines*. When the angle is less than 15°, the foot is considered normal; otherwise the deformation of the foot is considered to be of hallux valgus type (color figure online)

Foot problems prevail among almost all ethnic and age groups [6, 13, 16, 30]. Among all foot problems, three types of foot deformation occur with a high prevalence, namely pes valgus, hallux valgus and pes cavus [7, 38, 44]. Pes cavus and pes valgus both manifest with problems in the medial longitudinal arch, while hallux valgus is associated with a metatarsophalangeal angle greater than 15°. Figure 1 shows an illustration of the morphological structure of each of these deformations and the one of a normal foot. Each of the three deformations, if not recognized and treated early, will progress with complications (e.g. tibial and femoral stress fractures, metatarsalgia) [25, 40, 41]. Plantar pressure pattern can indicate the condition of the biomechanics of foot and ankle. It is widely used for diagnosis of foot health problems [1]. Considering the high prevalence of the mentioned three kinds of foot deformities nowadays, it is necessary to investigate new plantar pressure characteristics of them.

The human foot has two functions: weight-bearing and propulsion. The areas under the five metatarsal heads, hallux and calcaneus, are mainly load-bearing locations. Therefore, the peak and mean pressure values, the pressure–time integrals and the force under these areas are commonly used to analyze and compare plantar pressure patterns among foot types. Hallux valgus exhibits pressure peaks under the first metatarsal head and the hallux, while a normal foot exhibits peaks under the second and the third metatarsal heads [26]. The peak force under the hallux area of pes planus is higher than that of a normal foot [23]. Parameters as hallucial peak pressure, normalized peak pressure under the second metatarsus, hallucial maximum force, the maximum force under the second toe and its normalized value are all different between pes planus feet and pes cavus feet [20].

In the studies of foot function so far, the plantar pressures under three areas (the forefoot, midfoot and rearfoot) were also investigated. The peak pressure under the rearfoot in a pes cavus foot is different from the one in a normal foot. Also, the pressure–time integrals under the three areas as well as under the whole foot in the pes cavus foot are higher than those in a normal foot [5].

The CoP is the point of the plate where the ground reaction force applies. It can provide information about motion control [29] and reflects the result of the musculoskeletal interaction of lower extremities. Regarding the CoP characteristics of a normal foot, pes cavus and pes valgus feet, De Cock et al. [9] analyzed the medial–lateral displacement of CoP at the sub-stance phase, and found differences at the initial metatarsal contact, the forefoot flat sub-phase and the heel-off sub-phase. The trajectory of CoP in patients with hallux valgus shows that the patients’ big toes bear little or no weight [33]. There is a tendency for flat feet (i.e. pes valgus) the CoP pathway to get across the forefoot area with a shape closer to a straight line [18]. The Center of Pressure Excursion Index (CPEI), which derived from the center of pressure, can indicate the extent of foot pronation and supination during the stance phase. CPEI of pes planus feet is different from the ones of the rectus (normal) and pes cavus feet [20]. In previous studies, some discriminant features were selected from vertical ground reaction force (VGRF) and were used for recognition of pes valgus and a normal foot [4]. The peaks at the initial contact phase, as well as the peaks at the push-off phase reflect the differences between pes valgus and pes cavus [14].

All previous studies on the CoP pattern in the different foot types mainly focus on characteristics in the time domain and spatial domain. The discriminative parameters are extracted from one or several data points. For this reason, these parameters may easily be contaminated by noise. As pointed out in [34], sample entropy allows avoiding the influence of the noise when exploring time series. Furthermore, when it comes to gait analysis, foot data are obtained separately in each gait cycle, i.e. time series such as the CoP progression trajectory extracted for each gait cycle could be considered a relatively short time series. Sample entropy is very suitable to process such short time series [47]. Also, no effective parameters derived from the A/P component of CoP have been reported so far. Therefore, in this paper, we exploratively use the sample entropy to analyze the CoP displacement and aim to find effective parameters for foot function evaluation of the different foot types.

Sample entropy could be used to quantify the regularity and complexity of a data series, and for discovering changes in the underlying dynamic characteristics [36]. It finds wide applications for processing of physiological signals and human motion signals. Sample entropy was used for analyzing the CoP data obtained during still standing to investigate the relation between the CoP fluctuation [37], as well as for the comparison of attentional investment and postural sway fluctuations between children with cerebral palsy and healthy children [11].

Sample entropy can also be used to quantify regularity and complexity of CoP progression pattern [27]. We supposed that the differences in conditions of the four foot types would result in different entropy values due to the different patterns of CoP progression. Up until now, to our best knowledge, no study has reported the application of sample entropy to study the CoP trajectory pattern of the four foot types. Moreover, the parameters *m* and *r* for sample entropy calculation using CoP displacement data during the stance phase were not explored so far. In our previous research [27] we have used a sample entropy method to analyze the CoP variables of velocity and acceleration during the stance phase of the gait cycle. Later we hypothesized that statistical results might differ in the sample entropies of CoP variables of velocity, acceleration, and the CoP displacement. Thus, with the present study we aimed: (1) To find appropriate values of the parameters *m* and *r* to analyze CoP data during the stance phase using sample entropy. So far, there are no previous studies of this application of sample entropy. We expected that the optimal values of *m* and *r* parameters will differ from those used for CoP sway data analysis. (2) To investigate if there are differences in the sample entropy of CoP trajectory across the four foot types. (3) To compare the statistical characteristics of sample entropy CoP displacement component, velocity, and acceleration.

## Methods

### Experimental platform

**Fig. 2:**
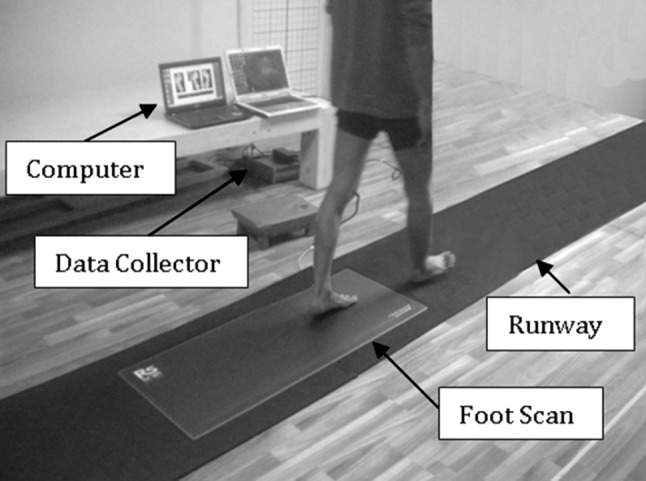
Experimental platform: it consists of Footscan^®^ sensor array, data collector, runway and computer

For acquisition of CoP data, including coordinates of CoP trajectory and the VGRF, we used a Footscan^®^ system. It is produced by RSscan International, Olen, Belgium and has dimensions of 1068 mm × 418 mm × 12 mm. Its plate contains 8192 sensors with a density of 2.6 sensors/cm^2^. It supports sampling rates of up to 500 Hz, depending on the operating mode. For our experiment, we chose to capture data from the Footscan^®^ plate at a sampling rate of 253 Hz, which is the maximum supported one when using the full spatial resolution (i.e. all available sensors). The plate was mounted in the middle of a walkway with a total length of 8 m (Fig. 2).

### Subjects and experimental protocol

We recruited 135 subjects in total (60 males, 75 females, foot size: 24.4 ± 1.5 cm, weight: 58.7 ± 15.5 kg, height: 162.5 ± 9.6 cm, age: 33.8 ± 14.8 years). Among them, there were 19 subjects with pes valgus, 40 with pes cavus, 36 with hallux valgus and 40 with normal feet. The experimental procedure was approved by the ethics committee of the Shenzhen Institutes of Advanced Technology. Prior to testing each subject signed an informed consent. Each participant was examined by a podiatrist. For each participant, a case report has been written which contains a detailed description of demographics, structure and functional condition of feet, lower limb and trunk, the types of both feet, and the gait pattern. A summary of the characteristics of all subjects is shown in Table 1. We did not include subjects with other musculoskeletal or neurological diseases or other kinds of foot problems such as diabetic foot and traumatism.

**Table 1:** Subject characteristics: mean value and standard deviation of age, height, weight and foot size of the subjects of each foot type group

	Normal *N* = 40	Pes valgus *N* = 19	Pes cavus *N* = 40	Hallux valgus *N* = 36	*p* value
Shoe Size (cm)	24.5 (1.5)	24.0 (1.9)	24.8 (1.5)	23.8 (1.1)	0.284
Weight (kg)	60.0 (12.1)	55.3 (24.4)	63.5 (16.5)	53.7 (9.4)	<0.001
Height (cm)	163.3 (8.6)	158.6 (14.1)	166.7 (9.1)	159.2 (6.3)	0.028
Age (years)	33.8 (13.2)	27.5 (16.9)	32.5 (13.1)	38.7 (16.1)	0.005

When adults walk in their daily life, they tend to do that at their preferred speed [19]. Thus, the biomechanical characteristics and the characteristic of plantar pressure for the preferred speed of walking are representative. To obtain plantar pressure data at a preferred speed, a mid-step protocol was used (i.e. we asked the subject to make at least three steps before and after contacting the Footscan [24, 28]). We instructed subjects to look forward and not to look down towards the pressure plate and the walkway during a trial. A trial was considered valid when it met the following criteria: (1) each subject has not suddenly changed his/her gait before and after accessing the plate i.e. there were no changes in the step length and cadence. (2) The plantar contact area was confined within the sensor area. (3) Each subject walked at a preferred speed. (4) For each subject, plantar data were acquired six times for each foot.

### Data analysis

**Fig. 3:**
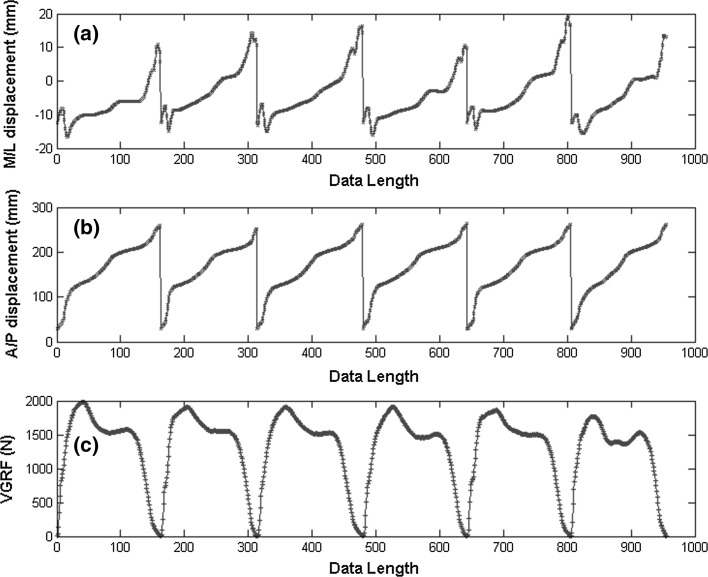
Illustration of CoP trajectory and the VGRF of CoP for the left foot during the stance phase. CoP can be decomposed into three components: **a** medial–lateral (M/L) displacement, **b** anterior–posterior (A/P) displacement and **c** VGRF of CoP

In this study, we used sample entropy to quantify several parameters of the four foot types. These parameters are the complexity and regularity of the M/L displacement and A/P displacement, and the VGRF of CoP. The investigated types of feet are pes valgus, pes cavus, hallux valgus and a normal foot. Figure 3 shows an illustration of the measured components. For each subject, the CoP data acquired from all measurements were spatially translated to get the same initial coordinates and then concatenated to a single time series.

Both sample entropy and approximate entropy can be used to analyze time series data. Approximate entropy is approximately equal to the negative natural logarithm of the conditional probability and involves self-match counting for time series. It is suitable to process time series with a length between 100 and 5000 [32]. Sample entropy derived from approximate entropy without self-match counting. Sample entropy is the negative natural logarithm of the conditional probability that subseries of length *m* that match pointwise within a tolerance *r* also match at the next point [31]. A low value of sample entropy indicates a low complexity of the time series.

The algorithm to calculate sample entropy (*SamEn*) is given below [31]. For a given time sequence *X*_*N*_ = [*x*_*1*_, *x*_*2*_, …, *x*_*n*_], construct subseries (i.e. vectors) of length *m* which are defined as *Xi* = (*x*_*i*_, *x*_*i*+*1*_, *…*, *X*_*i*+*m*-*1*_). The next step is to calculate the probability that any of the vectors will be similar to *X*_*i*_:

1 $$C_{i} = \frac{{n_{i} (m,r)}}{N - m + 1}$$

where *n*_*i*_ (*m, r*) stand for the number of vectors *X*_*j*_ that are similar to *X*_*i*_ with a constraint of $d(X_{i} ,X_{j} ) \le r$.

*d(X*_*i*_ *, X*_*j*_ *)* is defined as the maximal difference between vectors *X* and *Y* in their respective scalar components.

Then, calculate the average probability:

2 $$\varPhi (m,r) = \frac{1}{N - m + 1}\sum\limits_{i = 1}^{N - m + 1} {C_{i} (m,r)}$$

The same process is repeated for the subseries of length m + 1 to calculate $\varPhi (m + 1,r)$.

Finally, sample entropy is calculated as follows:

3 $$SamEn(X_{N} ,m,r) = - \ln \frac{\varPhi (m,r)}{\varPhi (m + 1,r)}$$

where ln is the natural logarithm.

To calculate the sample entropy of each of the CoP measurement variables, it is required to determine the parameters *m* and *r*. According to the recommendations given in [2] and [22] which apply to the general case, *m* can take a value of 1 or 2, and *r* can accept values between 0.1 and 0.25. However, since we acquired the CoP data during the stance phases, in our case *m* and *r* may differ from the recommended values. To determine the optimal values of *m* and *r* we used the method proposed in articles [35] and [22]. According to it, first, the conditional probability (CP) is to be calculated:

4 $$CP(m,r) = \frac{A(r)}{B(r)}$$

where *A*(*r*) and *B*(*r*) stand, respectively, for the number of matches of length *m* + *1* and *m* within tolerance *r*. Then, the variance of CP can be estimated as:

5 $$\sigma_{\text{CP}}^{2} = \frac{{{\text{CP}}(1 - {\text{CP}})}}{B} + \frac{1}{{B^{2} }}\left[ {K_{A} - K_{B} ({\text{CP}})^{2} } \right]$$

where *K*_*A*_ and *K*_*B*_ are, respectively, the number of pairs of matching templates of length *m* + *1* and *m* that overlap within tolerance *r*. The values *m* and *r* are determined by minimizing the maximum relative error *Q*(*m*, *r*) of SampEn and the CP estimate, which is defined as:

6 $${\text{Q}}({\text{m}},{\text{r}}) = \hbox{max} \left( {\frac{{\sigma_{\text{CP}} (m,r)}}{{{\text{CP}}(m,r)}},\frac{{\sigma_{\text{CP}} (m,r)}}{{ - \log ({\text{CP}}(m,r)){\text{CP}}(m,r)}}} \right)$$

With increasing the length of *m*, the accuracy and confidence of the sample estimate improve; with decreasing the *r* value, the discriminative ability of the sample estimate also improves [22]. Therefore, we should choose *m* value as large as possible and r value as small as possible. This metric simultaneously penalizes CP near 0 and 1 and it is a trade-off between accuracy and discriminative capability. In our analysis, we set the maximal relative error criterion to be less than 0.05 which corresponds to a case when the 95 % confidence interval of the sample entropy estimate is maximum 10 % of its value.

### Statistical analysis

To research the relation between the different factors (including the height, weight, shoe size, age and the length of data series) and the corresponding sample entropy values, we performed tests of between-subjects effects. We then used the factors that have an effect on the measured variables as covariates in the subsequent statistical analysis. We explored two cases: calculating the sample entropy using the raw data of CoP components, as well as calculating it for normalized CoP components. For the case of non-normalized CoP data, we performed statistical analysis as follows. To achieve equality of error variance, we log-transformed the measured values for M/L and VGRF of both side feet for the four feet groups. We then performed an analysis of covariance on the sample entropy of A/P and VGRF with pairwise comparisons with Bonferroni adjustment. Because of the inequality of variance of sample entropy of the M/L displacement of the left feet, for the statistical analysis, we applied a Kruskal–Wallis test with pairwise comparison. Analysis of covariance was performed on the M/L displacement.

To investigate if using normalized CoP data could lead to a better result, we normalized the M/L displacement by the foot width, the A/P displacement by the foot length, and the VGRF by the subject weight. Then, we used sample entropy to quantify the normalized data. Before the statistical analysis, to achieve equality of error variance we log-transformed the quantified A/P variables and VGRF values of the left foot. Then we performed analysis of covariance on the sample entropy of the A/P displacement and VGRF values with pairwise comparisons with Bonferroni adjustment. All statistics were calculated using SPSS 20.0 and *p* < 0.05 was taken as a significant level.

## Results

### Parameters *m* and *r* for sample entropy calculation

**Fig. 4:**
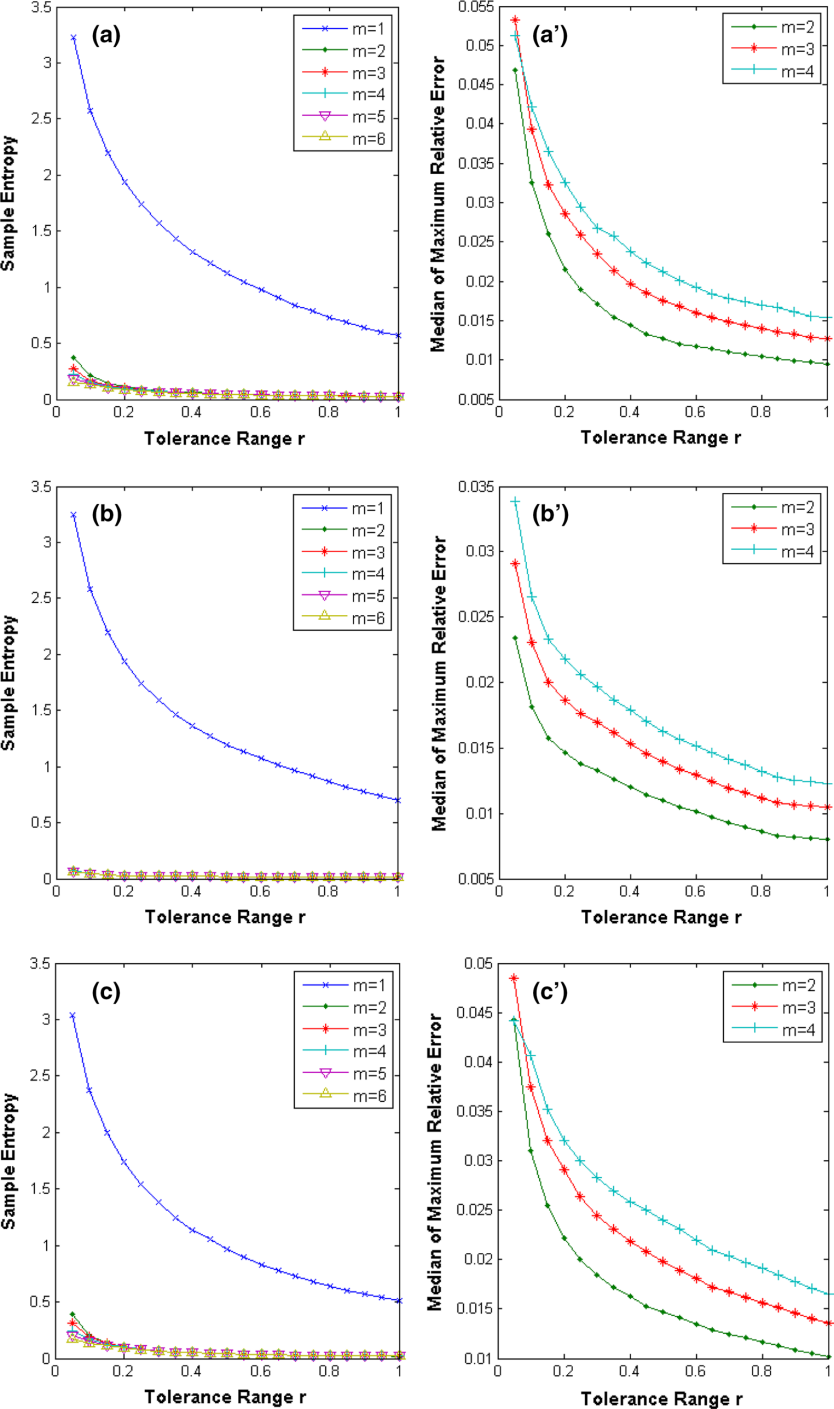
Determination of parameters *m* and *r*. The *left column* shows sample entropy computed for *m* = 1–6 and *r* ranging from 0.05 to 1 with a step of 0.05. **a**–**c** Sample entropy of M/L, A/P and VGRF, respectively. The *curves* for m ≥ 3 almost converge in (**a**), as well as the curves for *m* ≥ 2 and *m* ≥ 3 in (**b**) and (**c**), respectively. The *right column* shows the computed maximum relative error for *m* = 2–4 and *r* ranging from 0.05 to 1 with a step of 0.05. For all measured variables, the maximum relative error is below 0.05 when *r* = 0.1

In Fig. 4 we illustrate the optimal values of parameters *m* and *r* for the left foot, where *m* and *r* are determined using the non-normalized CoP data. The median sample entropy of M/L variables converges when *m* ≥ 3 for almost all *r* values. The median sample entropy of the A/P and VGRF variables converges when *m* ≥ 2. When the median of the maximum relative error is below 0.05, each r value is 0.1 for all measured variables. Therefore, we computed the sample entropy values using *m* = 3, *r* = 0.1 for M/L time series, and *m* = 2, *r* = 0.1 for both A/P and VGRF time series of left feet and right feet. For the normalized CoP data, the optimal values of *m* and *r* are the same as those used for the non-normalized CoP data.

### Sample entropy characteristics for the four foot types

We analyzed the effects of the data length, as well as the subjects’ weight, height, age, gender, and shoe size on the sample entropy of the measured variables for both the case of non-normalized CoP variables and for normalized CoP variables. The results are given in Table 2. Our results indicate that each of the variables is influenced by at least one of the factors.

**Table 2:** Comparison tests of between-subjects effects for the non-normalized data and normalized data

Parameter	Non-normalized data						Normalized data					
	Weight	Height	Data length	Age	Gender	Shoe size	Weight	Height	Data length	Age	Gender	Shoe size
	*F* (*p* value)	*F* (*p* value)	*F* (*p* value)	*F* (*p* value)	*F* (*p* value)	*F* (*p* value)	*F* (*p* value)	*F* (*p* value)	*F* (*p* value)	*F* (*p* value)	*F* (*p* value)	*F* (*p* value)
Left foot												
M/L	**4.451 ** **(0.037)**	0.012 (0.912)	1.113 (0.293)	2.742 (0.100)	0.027 (0.869)	0.008 (0.929)	0.013 (0.910)	**3.935** **(0.049)**	3.678 (0.057)	**12.743** **(0.001)**	**18.031** **(<0.001)**	**5.819** **(0.017)**
A/P	0.504 (0.479)	**13.931** **(<0.001)**	0.009 (0.926)	2.909 (0.091)	0.147 (0.702)	0.317 (0.574)	1.384 (0.242)	2.911 (0.090)	**12.820** **(<0.001)**	**11.670** **(0.001)**	3.465 (0.065)	0.839 (0.361)
VGRF	**4.296** **(0.040)**	1.363 (0.245)	**18.595** **(<** **0.001)**	3.636 (0.059)	**5.529** **(0.020)**	1.796 (0.183)	1.684 (0.197)	2.510 (0.116)	**11.731** **(0.001)**	2.906 (0.091)	**5.956** **(0.016)**	2.435 (0.121)
Right foot												
M/L	**4.971** **(0.028)**	0.004 (0.949)	1.104 (0.295)	**4.794** **(0.030)**	0.061 (0.806)	0.072 (0.789)	0.075 (0. 785)	**11.783 ** **(0.001)**	0.039 (0.843)	**6.116** **(0.015)**	**8.414** **(0.004)**	0.426 (0.515)
A/P	0.022 (0.882)	**5.534 ** **(0.020)**	0.004 (0.952)	0.827 (0.365)	2.816 (0.096)	1.716 (0.193)	0.8930 (0.347)	1.144 (0.287)	3.360 (0.069)	**17.721 ** **(<** **0.001)**	**5.947** **(0.016)**	4.622 (0.033)
VGRF	**5.018 ** **(0.027)**	3.713 (0.056)	**5.928** **(0.016)**	**11.830** **(0.001)**	**6.823** **(0.010)**	0.407 (0.525)	**5.859** **(0.017)**	3.363 (0.069)	2.024 (0.157)	**9.861** **(0.002)**	3.863 (0.052)	0.129 (0.720)

Bold values represent statistical difference with *p* < 0.05

In the above table, the following abbreviations were used: *STD* standard deviation, *M/L* medial–lateral displacement, *A/P* anterior–posterior displacement, V*GRF* vertical ground reaction force

There is no difference in M/L measured values of the left foot with (*F* = 0.596, *p* = 0.427). Sample entropy of M/L measured values of the right foot are not significantly different among the four foot types (*F* = 0.558, *p* = 0.644). In Table 3, we illustrate the means and standard deviations of the sample entropy for the A/P and VGRF measurements of the both-side feet. We did not find a difference for the VGRF across the four foot types. The sample entropy of the non-normalized A/P displacement of pes valgus is different from the ones of the normal foot and hallux valgus (for the left foot *F* = 4.868, *p* = 0.003 and for the right foot *F* = 5.162, *p* = 0.002, respectively).

**Table 3:** Mean, standard deviation and pairwise comparisons of sample entropy for the non-normalized CoP components between the normal foot, pes cavus, pes valgus, and hallux valgus

	Mean (STD)				*p*
	Normal foot	Pes valgus	Hallux valgus	Pes cavus	
Left foot					
A/P	−3.213 (0.185)^b^	−2.929 (0.397)^a,c^	−3.134 (0.235)^b^	−3.122 (0.288)	0.003
VGRF	−1.720 (0.295)	−1.572 (0.541)	−1.616 (0.296)	−1.659 (0.373)	0.549
Right foot					
A/P	−3.239 (0.205)^b^	−2.948 (0.369)^a,c^	−3.147 (0.218)^b^	−3.132 (0.305)	0.002
VGRF	−1.705 (0.304)	−1.493 (0.437)	−1.592 (0.277)	−1.688 (0.384)	0.617

Prior to the statistical analysis, variables were log-transformed

In the above table, the following abbreviations were used: *STD* standard deviation, *A/P* anterior–posterior displacement, *VGRF* vertical ground reaction force

^a,b,c,d^ Significantly different from normal foot (a), pes valgus (b), hallux valgus (c) and pes cavus (d), respectively

We provide the result of statistical analysis based on normalized CoP data in Table 4. The sample entropy of M/L variables of the right foot is different between the normal foot and pes cavus (*F* = 2.815, *p* = 0.042). We did not find any difference in the sample entropy of the other variables.

**Table 4:** Mean, standard deviation and pairwise comparisons of sample entropy of normalized CoP components between the normal foot, pes cavus, pes valgus, and hallux valgus

	Mean (STD)				*p*
	Normal foot	Pes valgus	Hallux valgus	Pes cavus	
Left foot					
M/L	−4.153 (0.225)	−3.999 (0.320)	−4.180 (0.292)	−4.141 (0.230)	0.590
A/P	−3.937 (0.166)	−3.802 (0.308)	−3.876 (0.212)	−3.838 (0.219)	0.163
VGRF	−0.060 (0.266)	−0.010 (0.502)	0.058 (0.249)	−0.018 (0.340)	0.614
Right foot					
M/L	−4.273 (0.245)^d^	−4.021 (0.364)	−4.193 (0.275)	−4.145 (0.278)^a^	0.042
A/P	−3.955 (0.182)	−3.791 (0.296)	−3.883 (0.184)	−3.872 (0.237)	0.089
VGRF	−0.034 (0.292)	0.108 (0.324)	0.048 (0.236)	−0.023 (0.372)	0.619

Prior to statistical analysis, log-transform was performed

In the above table, the following abbreviations were used: *STD* standard deviation, *M/L* medial–lateral displacement, *A/P* anterior–posterior displacement, *VGRF* vertical ground reaction force

^a,b,c,d^ Significantly different from normal foot (a), pes valgus (b), hallux valgus (c) and pes cavus (d), respectively

## Discussion

In this study, sample entropy was used to quantify complexity and regularity of M/L and A/P displacements, and the VGRF of CoP during the stance phase, respectively. There was statistically significant difference in the sample entropy between the normal foot and pes valgus, as well as between the normal foot and hallux valgus for the A/P measurements of both-side feet. After normalizing the CoP data, we found that the sample entropy of M/L measurement is different between the normal foot and pes cavus foot.

### Between-subjects effects of subjects’ characteristics

The parameters under exploration in the present work are all dependent on the personal characteristics of the subject. Therefore, we hypothesized that subject characteristics might influence the sample entropy results. We investigated the impact of several factors on the sample entropy results for both non-normalized and normalized data. For normalized data, we found that weight, height, data length, age, and gender had an effect on the sample entropy of at least one CoP component. Below we provide a discussion of the influence of each parameter on the sample entropy for non-normalized data.

The weight had an impact on the sample entropy of M/L displacement and VGRF. This observation is partly consistent with a previously obtained result of analysis of temporal-spatial parameters. For instance, obese subjects exhibit greater A/P and M/L displacement, and increased ground reaction force [10].

In the present study, we found that height had an influence on sample entropy of A/P displacement. A subject with a lower height has a lower center of mass, which results in a more stable gait [17]. Thus, the CoP of these subjects might be more regular. Hence, the height has an effect on the sample entropy of the CoP components.

After performing a test of between-subjects effects, we found that the data length had a significant influence on the entropy value of VGRFs. For a given number of stance phases, longer total duration of the stance phase (i.e. including all cycles) means a lower walking speed. Variable walking speed will lead to a corresponding variation of foot biomechanics [45]. The butterfly diagrams constructed using VGRF are different for different walking speeds [12]. Thus, the differences in the walking speed will lead to possibly different values when calculating the sample entropy for VGRF.

Ageing is always accompanied by a decrease in entropy [42]. Consequently, the between-subjects effects of age should be tested. We performed such a test and found that age had an effect on the sample entropy of VGRF and M/L displacement of the right foot.

In our study, the gender as one of the factors had an effect on the sample entropy of VGRF. The possible reason is that women’s walking speed is lower than that of men for all age ranges [39]. We already discussed above the walking speed as a factor. Also, the patterns of plantar pressure distribution are different between the male and female groups [8]. This difference may determine corresponding gender-dependent differences in the sample entropy of the CoP components.

When we compared the influence of the same factors on the sample entropy of each CoP component between the left and right foot, we found that the between-subjects effects were different. For example, age had an effect on the sample entropy of the M/L displacement of the right foot, but not on the one of the left foot. This phenomenon could be explained with the functional asymmetry of the left and the right foot. Also, the between-subjects effects for the normalized data and non-normalized data are different. The normalization did not remove the effects of all factors. Future studies are needed to explore in detail the influence of the different subject characteristics as factors in the sample entropy calculation.

### Determination of functional differences between the four foot types using sample entropy analysis

The morphological differences between the four foot types determine different CoP progression patterns. The combination of different movement patterns of dorsiflexion and plantarflexion, eversion and inversion, abduction/adduction and the differences in anatomical structures, result in different application points of CoP in the A/P direction. The pathway of CoP during the stance phase will contain information about its progression patterns.

Regarding the analysis of the CoP displacement of different foot types, to our best effort, we have not found any parameter extracted from the A/P displacement of CoP in other studies. CPEI (which was derived from the CoP displacement) and VGRF were investigated in previous studies [3, 14, 15, 43]. The M/L displacement can mainly indicate the movement characteristics for eversion and inversion in the transverse plane during the stance phase. The M/L displacement of CoP at all key events and CPEI can indicate the foot function in M/L direction. We found that sample entropy of the normalized M/L displacement was statistically different between the normal foot and pes cavus foot, where its value for the normal foot was lower than the one for pes cavus foot. This observation indicates that in the M/L direction the CoP of the pes cavus foot is more irregular than the one of a normal foot. It also indicates that people with pes cavus have unstable gait in the M/L direction. These facts could contribute to the explanation why lateral injuries mainly occur in pes cavus group, although additional investigation would be needed to determine if results for normal walking speed would correlate with these for other kinds of gait like running [46].

Besides eversion and inversion, during the stance phase, the foot also performs dorsiflexion and plantar flexion. Dorsiflexion and plantar flexion during the stance phase mainly contribute to the A/P displacement. However, so far, to our best knowledge, no study explored valuable parameters extracted from CoP A/P displacement using linear methods. In this study, we found significant differences in the sample entropy of the non-normalized A/P displacement between the normal foot and pes valgus foot, as well as between the normal foot and hallux valgus foot. These differences may indicate that foot function during dorsiflexion/plantarflexion is different between these three foot types. Regarding the measured A/P variables, the ones of the normal foot type exhibited the lowest sample entropy. The A/P variables of pes valgus and pes cavus had the larger sample entropy. The results also show that CoP trajectory of the normal foot in A/P direction exhibits more regular and less fluctuating behaviour during stance phases when compared with the ones of pes valgus and pes cavus feet. Less regularity and more fluctuation mean that a subject with pes valgus has a lower gait stability. Here may lie one of the possible reasons why a subject with pes valgus would spend more energy [3, 21] and would easily get tired when compared with a subject with normal feet [14].

As to the comparison of sample entropy between the four types of feet, there is no difference between pes planus and pes cavus feet. That observation follows from the fact that sample entropy quantification does not allow to discriminate the irregular fluctuations of CoP in A/P direction of these two types of feet. Indeed, some parameters extracted from plantar pressure data are different between pes planus (valgus) and pes cavus feet. However, for some of the parameters, e.g. the peak pressure at the metatarsal head 2 [20], there is a difference only between pes valgus and a normal foot. Hence, for the evaluation of foot function of pes cavus and pes valgus, the appropriate parameters should be chosen.

### Comparative analysis of sample entropy of CoP variables

We found a statistical difference in the sample entropy of normalized A/P displacement between the normal foot and pes valgus, as well as between the normal foot and pes cavus. On the other hand, the sample entropy of A/P velocity of pes cavus of both-side feet appeared to be different from that of the other types of feet [27]. Therefore, the sample entropy of CoP variable of A/P displacement was different from the one of A/P velocity.

Regarding the sample entropy of CoP variables of acceleration, in the case of left feet, there was no difference among the four types of feet. However, when comparing the CoP variables of the right foot, the sample entropy of A/P variable of the normal foot is different from the ones of pes valgus and pes cavus feet. Besides, there is a difference in the sample entropy of A/P acceleration between hallux valgus and pes cavus feet [27].

The conclusion from the pairwise comparison is that there exist a difference in the statistical characteristics of sample entropy of CoP displacement, velocity, and acceleration. The sample entropy of CoP displacement, velocity and acceleration can be used together for evaluation of the foot function.

### Future work

A 3D force plate can indicate the change of the force and moment in A/P, M/L and vertical directions. If such a device were combined in this study, more information about dynamic progression patterns would be explored, and it would be possible to obtain more discriminative features of sample entropy.

In this study, all samples were roughly classified into four groups. In fact, each group of abnormal feet can be divided into subgroups according to structural or pathological abnormities. For example, pes cavus consists of two subgroups: idiopathic and neurogenic, and there are different patterns of plantar pressure distribution between these two subgroups [5]. As a result, entropy values between subgroups might differ. Therefore, considering more subgroups will lead to a more informative result.

In contrast to the analysis of anatomical structures using medical imaging or analysis of CoP in the time domain, the proposed method could not explain, in an easily understandable way, e.g. kinetic and kinematic distortion degree of the musculoskeletal system and the displacement range and velocity of supination or pronation. However, it could be used to study foot condition as a whole, from another perspective.

## Conclusions

The main novelty of the proposed method is the suggestion, when investigating foot function, to take into account dynamic characteristics of CoP progression that contain the dynamic information about walking pattern. We used sample entropy to quantify the complexity of CoP, which was decomposed into three components: M/L displacement, A/P displacement and VGRF of CoP.

In this work, in terms of sample entropy analysis of CoP data collected during the stance phase, the optimal values of parameters *m* and *r* were different from the values recommended in the general case. The sample entropies of the non-normalized A/P displacement for the left foot and the right foot, respectively, were different between the normal foot and pes valgus foot, as well as between the normal foot and hallux valgus foot. The measured values of the normalized M/L displacement of the right foot were different between the normal foot and pes cavus foot. These results could potentially be used as a reference to study the foot function. They also indicate that it is feasible to analyze CoP data during the stance phase using sample entropy.
